# The Novel Nature Microtubule Inhibitor Ivalin Induces G2/M Arrest and Apoptosis in Human Hepatocellular Carcinoma SMMC-7721 Cells In Vitro

**DOI:** 10.3390/medicina55080470

**Published:** 2019-08-12

**Authors:** Fangyuan Liu, Shiqi Lin, Caiyun Zhang, Jiahui Ma, Zhuo Han, Fujuan Jia, Weidong Xie, Xia Li

**Affiliations:** 1 College of Marine Science, Shandong University, Weihai 264209, China; 2 School of Pharmaceutical Sciences, Shandong University, Jinan 250012, China; 3 The Key Laboratory of Chemistry for Natural Product of Guizhou Province and Chinese Academy of Science, Guiyang 550002, China

**Keywords:** Ivalin, *carpesium divaricatum*, HCC, microtubule, cell cycle arrest, apoptosis

## Abstract

*Background and Objectives*: Microtubules are an attractive target for cancer chemotherapy. Previously, we reported that Ivalin exhibited excellent anti-migration and anti-invasion activities in human breast cancer cells. Here, we examined the microtubule inhibition effect of Ivalin in human hepatocellular carcinoma SMMC-7721 cells. *Materials and Methods*: We used the 3-(4,5-dimethylthiazol)-2,5-diphenyltetrazolium bromide (MTT) assay to evaluate the cell proliferation effect of Ivalin and flow cytometry analysis to detect the apoptotic and cell cycle arrest effects of Ivalin. Immunofluorescence staining was used to measure the effect of Ivalin on the cytoskeleton network, and Western blotting was used to detect the expression levels of Bax, Bcl-2, Cdc2, phosphor-Cdc2, Cdc25A, Cyclin B1, and tubulin. *Results*: Ivalin induced cell cycle G2/M arrest and subsequent triggered apoptosis in human hepatocellular carcinoma SMMC-7721 cells. Furthermore, microtubules were shown to be involved in Ivalin-meditated apoptosis. In this connection, Ivalin treatment suppressed cellular microtubule network formation by regulating microtubule depolymerization. Moreover, Western blotting revealed Cdc25A and Cyclin B1 were upregulated in Ivalin-meditated cell cycle arrest. Subsequently, the induction of Bax (a proapoptotic protein) and reduction of Bcl-2 (an anti-apoptotic protein) expression were observed in Ivalin-treated SMMC-7721 cells. *Conclusion*: Ivalin induced microtubule depolymerization, then blocked cells in mitotic phase, and eventually resulted in apoptosis in SMMC-7721 cells. Collectively, these data indicate that Ivalin, acting as a novel inhibitor of microtubules, could be considered as a promising lead in anticancer drug development.

## 1. Introduction

Cancer is a major public health problem globally, and one of the most common primary tumors in adults is hepatocellular carcinoma (HCC), with a high death rate worldwide [1]. Many traditional treatment strategies, including surgical resection, chemotherapy, radiofrequency ablation therapy, and liver transplantation have been adopted because of the development of advanced treatments in recent years [2,3]. The total cure rate of HCC is difficult to improve, due to its high recurrence rate, rapid proliferation, and easy drug resistance [4]. Therefore, to develop new promising therapies for the treatment of HCC becomes necessary.

Highly dynamic mitotic-spindle microtubules have been proven to be some of the most successful targets for anticancer therapy [5]. Microtubules consist of α-tubulin and β-tubulin heterodimers [6]. Microtubules are extremely significant in the process of mitosis [7]. Mitosis in most cancer cells progresses rapidly, during which cell cutting into two daughter cells with separated duplicated chromosomes and highly dynamic microtubules in the spindle are required for all stages of mitosis [5,8]. Reports also confirmed that the usage of drugs with microtubule dynamic suppression effects, such as *Vinca* alkaloids and paclitaxel, may block mitosis and kill cancer cells [9,10]. These important functions of microtubules in mitosis and cell division make it an important target for anticancer therapies.

Many microtubule inhibitors have been used in great success with the disruption of microtubule functions by binding to the tubulin subunit, inducing mitotic arrest and subsequent apoptosis [11,12]. However, the frequently treatment of microtubule inhibitors leads to multidrug resistance (MDR), causing clinical treatment failures [13,14]. For this reason, finding new microtubule inhibitors becomes meaningful to the clinical trials. Ivalin, a eudesmane-type sesquiterpene compound isolated from the Chinese herb *Carpesium divaricatum*, showed excellent anti-migration and anti-invasion activities in human breast cancer cells in our previous studies [15,16]. However, the effects of Ivalin on microtubule dynamics have not been reported to date. In this study, we suggest that Ivalin could serve as an effective anti-microtubule agent with cell cycle arrest activity by microtubule depolymerization, which would finally result in apoptosis in hepatocellular carcinoma cell lines.

## 2. Materials and Methods

### 2.1. Chemicals and Reagents

Ivalin (>98%) was isolated from *Carpesium divaricatum* and identified by ESI-MS, ^1^H, and ^13^C NMR data [16]. Compound were dissolved at 10 μM in dimethyl sulfoxide (DMSO) as a stock solution and diluted to desired concentrations according to the research requirement. 3-(4,5-dimethylthiazol)-2,5-diphenyltetrazolium bromide (MTT) and 4,6-diamidino-2-phenylindole (DAPI) were purchased from Sigma-Aldrich Corp. (St. Louis, MO, USA). Antibodies against phosphor-Cdc2, Cdc2, Cdc25A, Cyclin B1, Bcl-2, and Bax were purchased from Cell Signaling Technology (CST, Inc., Beverly, MA, USA). Tubulin antibody was purchased from Proteintech Group, Inc. (Chicago, IL, USA). Glyceraldehyde-3-phosphate dehydrogenase (GAPDH) antibody was purchased from Abcam, Inc. (Cambridge, MA, USA). We bought the Annexin V–fluoresceine isothiocyanate (FITC) Apoptosis Detection Kit from BD Biosciences (San Jose, CA, USA). Propidium iodide (PI) and RIPA lysis buffer were obtained from the Beyotime Institute of Biotechnology (Shanghai, China). Vincristine (VCR) was purchased from Shenzhen Main Luck Pharmaceuticals Inc. (Shenzhen, China).

### 2.2. Cell Lines and Cell Culture

Human hepatocellular carcinoma cell lines HepG-2, Plc-prf-5, Hu-7, and SMMC-7721 and normal human hepatocyte cell line HL7702 were purchased from the Shanghai Institute for Biological Sciences (SIBS, Shanghai, China); we cultured all these cell lines according to the suppliers’ instructions.

### 2.3. MTT Assay

Cytotoxicity effect of Ivalin was evaluated by MTT assay. We seed the cells in 96-well plates at a density of 5 × 10^3^ cells per well. After 24 h of incubation, we treated the cells with Ivalin at various concentrations ranging from 0 to 50 μmol/L. Equal volumes of DMSO were used as the negative control. After 24–72 h of continuous culturing, 15 μL MTT (5 g/L) were added for 4 h incubation. Then, 150 μL DMSO were added after removing the medium, and the absorbance was read by a microplate reader at 570 nm. IC_50_ values (concentration resulting in 50% inhibition of cell growth) were calculated compared with the negative control, which was considered to have 100% cell survival. 

### 2.4. Immunofluorescence Staining

We seeded the cells on glass over slips and treated them with 0 to 8 μmol/L Ivalin for 24 h. Immunofluorescence staining was performed as previously described [17].

### 2.5. Western Blot Analysis

We exposed the cells to indicated concentration of Ivalin for 24 h, then the protein expressions were analyzed by Western blotting which we carried out as previously described [17].

### 2.6. Cellular Tubulin Polymerization Assays

We treated the cells with 4 μmol/L Ivalin or 0.1 μmol/L Vincristine and harvested the cells in the RIPA lysis buffer. Then the soluble tubulin fraction (supernatant) and the insoluble fraction (precipitate) were separated by centrifugation at 120,000× *g* and determined using Western blot analysis as mentioned above.

### 2.7. Flow Cytometry Analysis of Apoptosis

In order to measure the Ivalin-induced apoptosis effect in SMMC-7721 cells, we performed the flow cytometry analysis using the Annexin V-FITC apoptosis detection kit. We seeded the cells in 6-well culture plates at a density of 5 × 10^4^ cells per well and exposed them to different concentrations of Ivalin for 48 h, followed by washing with ice-cold PBS and trypsinization. According to the manufacturer’s instructions, each sample was co-stained with Annexin V–PI and the apoptotic ratio was analyzed by flow cytometry (Becton Dickinson FACScan, San Jose, CA, USA). Each sample was measured in triplicate experiments independently.

### 2.8. Cell Cycle Distribution

We treated the cells with varying concentration of Ivalin for 0–48 h. Cells were collected with trypsin and washed twice with PBS, then fixed with 75% alcohol for 12 h at 4 °C. After the fixation, cells were stained with propidium (PI), which was contained with 2% RNase A according to the manufacturer’s instructions. Flow cytometry was used to evaluate the cell cycle distribution and the Modfit program (Becton Dickinson FACScan, San Jose, CA, USA) was used to analyze the data.

### 2.9. Statistical Analysis

All experiments were performed at least three times. All data were represented as the mean ± standard deviation and analyzed using one-way analysis of variance (ANOVA) followed by SPSS 16.0 (SPSS Inc., Chicago, IL, USA). * *p* <0.05; ** *p* <0.01; *** *p* <0.001 were considered statistically significant.

## 3. Results

### 3.1. Cytotoxicity of Ivalin in Human Hepatocellular Carcinoma Cell Lines

**Figure 1:**
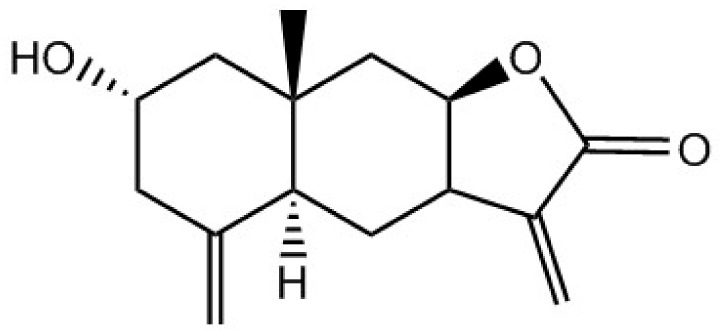
Chemical structure of Ivalin [15].

**Figure 2:**
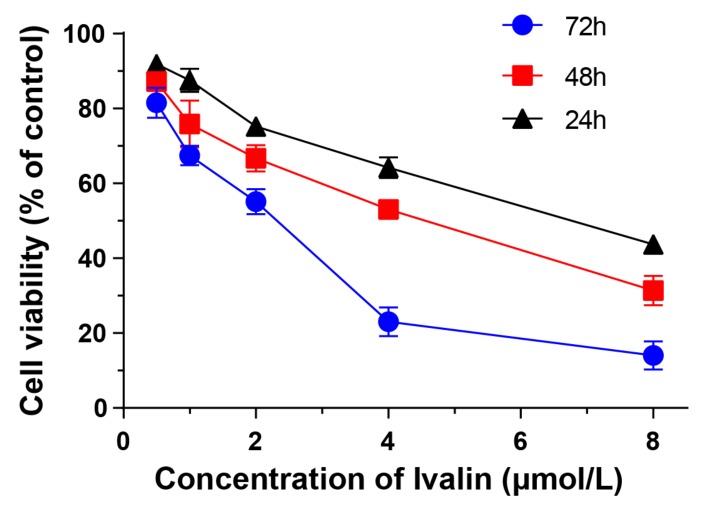
Dose- and time- dependent anti-proliferative activity of Ivalin on SMMC-7721 cells. Cells were treated with Ivalin (0 to 8 μmol/L) for 24 h to 72 h and the IC_50_ values were detected via MTT assay.

First, we initially performed MTT colorimetric assay in several subtypes of human hepatocellular carcinoma cell lines and normal human hepatocyte cell lines to assess the cytotoxic effect of Ivalin (Figure 1). As shown in Table 1, we found that Ivalin displayed a great sensitivity in SMMC-7721 cells, and the cytotoxicity in normal human hepatocyte cell lines HL7702 was lower. The IC_50_ values of Ivalin for 48 h treatment were 5.45 ± 0.13 μmol/L, 4.34 ± 0.10 μmol/L, 13.01 ± 0.42 μmol/L, 11.33 ± 1.00 μmol/L, and 25.86 ± 0.87 μmol/L for HepG-2, SMMC-7721, Hu-7, Plc-prf-5, and HL7702 cells, respectively. Moreover, Ivalin treatment dose- and time- dependently inhibited the proliferation of SMMC-7721 cells. The IC_50_ values of 24–72 h Ivalin treatment on SMMC-7721 cells were 7.39 ± 0.36 μmol/L, 4.34 ± 0.10 μmol/L, and 2.27 ± 0.13 μmol/L, respectively (Figure 2). Therefore, we chose SMMC-7721 cells to further explore the anticancer effects and mechanisms of Ivalin.

### 3.2. Effects of Ivalin on Apoptosis in SMMC-7721 Cells

**Figure 3:**
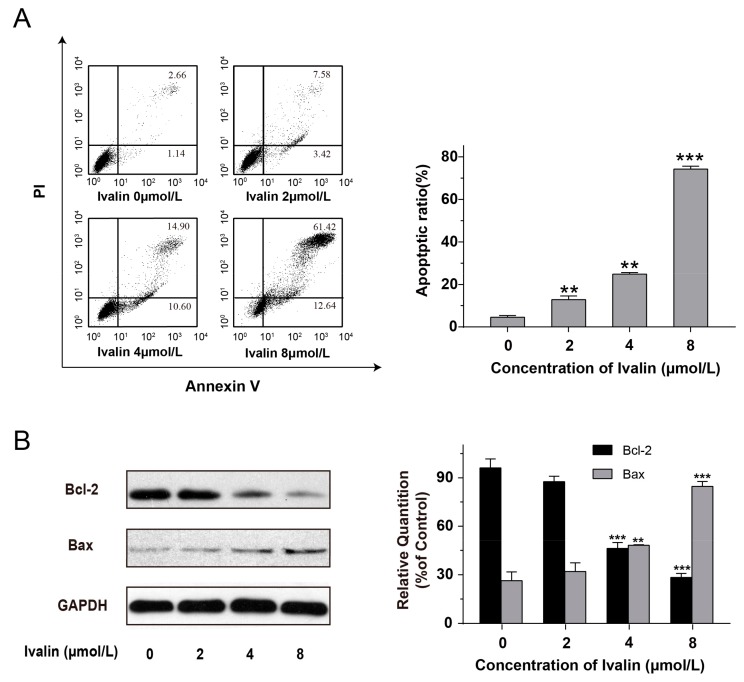
Ivalin-induced apoptosis was observed in SMMC-7721 cells. (**A**) Quantification of Ivalin-induced apoptosis in SMMC-7721 cells using flow cytometric analysis; (**B**) The protein expressions were measured by Western blot after 0 to 8 μmol/L Ivalin treatment. Results were obtained from three independent experiments. ** *p* < 0.01; *** *p* < 0.001 *vs* the control group.

Next, we quantified the extent of Ivalin-induced apoptosis. We co-stained the cells with Annexin V–PI and subsequently analyzed by flow cytometry. During the early apoptosis, cells could be stained with Annexin V, which binds to the phosphatidylserine on the outer surface of the plasma membrane. Propidium iodide (PI) could stain the late-stage apoptotic cells and necrotic cells by binding to DNA. In this connection, annexin V can then be used to specifically target and reflect apoptotic cells. Figure 3A showed a dose-dependent increase of the proportion of cells in apoptotic situation in the Ivalin-treated group of 3.80%, 11.00%, 25.50%, and 74.06% for 0 μmol/L, 2 μmol/L, 4 μmol/L, and 8 μmol/L of Ivalin treatment, respectively. Moreover, Western blot analysis showed that Ivalin treatment upregulated the ratio of Bax/Bcl-2 (Figure 3B). These data indicated that Ivalin triggered apoptosis in SMMC-7721 cells.

### 3.3. Effects of Ivalin on Cell Cycle in SMMC-7721 Cells

**Figure 4:**
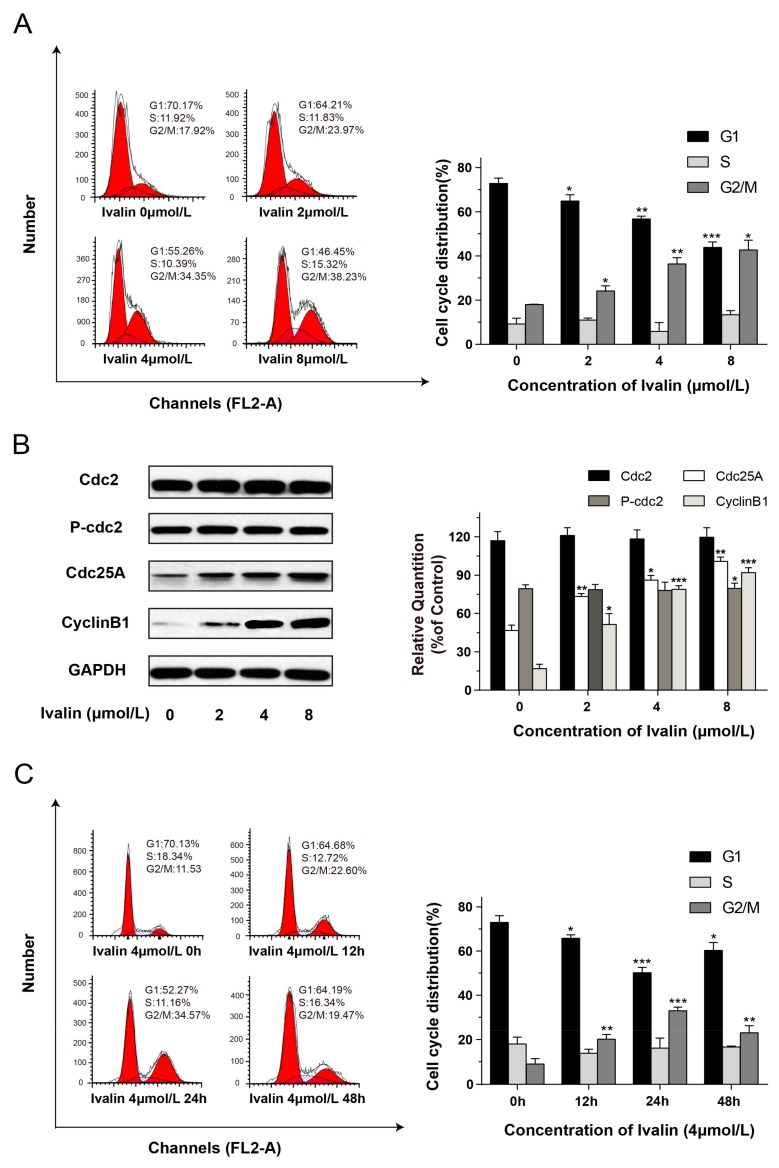
Ivalin dose- and time- dependently arrested cells in G2/M phase. (**A**) Flow cytometry was used to assessed the cell cycle distribution after 0 to 8 μmol/L Ivalin treatment; (**B**) The protein expressions of M-phase promoting factors (phosphor-Cdc2, Cdc2, Cyclin B1, and Cdc25A) were measured by Western blot after 0 to 8 μmol/L Ivalin treatment; (**C**) Cell cycle patterns of SMMC-7721 cells treated with 4 μmol/L Ivalin for 0 h to 48 h by flow cytometric analysis. Results were obtained from three independent experiments. * *p* < 0.05; ** *p* < 0.01; *** *p* < 0.001 *vs* the control group.

We used flow cytometry to detect the effects of Ivalin on cell cycle progression for a detailed mechanism survey. Figure 4A showed a typical G2/M arrest in SMMC-7721 cells after Ivalin treatment in a dose-dependent manner. The proportion of G2/M phase arrested cells significantly increased to 38.23% with 8 μmol/L Ivalin treatment for 24 h.

It is noted that Cdc2/Cyclin B1 complexes and Cdc25 were originally known as the M-phase promoting factors (MPF). The activation of the Cdc2/Cyclin B1 complex is considered to be the switch to mitosis in all eukaryotic cells, and Cdc25 activates the complex to trigger the initiation to mitosis [18]. Furthermore, we performed Western blotting to analyze these M-phase promoting factor expressions (phosphor-Cdc2, Cdc2, Cyclin B1, and Cdc25A). Results showed that Ivalin treatment led to the induction in Cdc25A and Cyclin B1 expression, while the expression of phosphor-Cdc2 and Cdc2 remained unchanged, which confirmed cells arrested in M phase rather than G2 phase after exposure to varying concentration of Ivalin (Figure 4B).

We also analyzed the cell cycle distribution of SMMC-7721 cells for 0 to 48 h treatment with 4 μmol/L Ivalin. As shown in Figure 4C, there was a remarkable accumulation of SMMC-7721 cells in G2/M phase for 24 h treatment of Ivalin (34.57%) but this decreased to 19.47% after 48 h of continuous treatment (Figure 4C). The above results suggest that the subsequent apoptosis may follow persistent mitotic arrest.

### 3.4. Effects of Ivalin on the Microtubule Tissue of SMMC-7721 Cells

**Figure 5:**
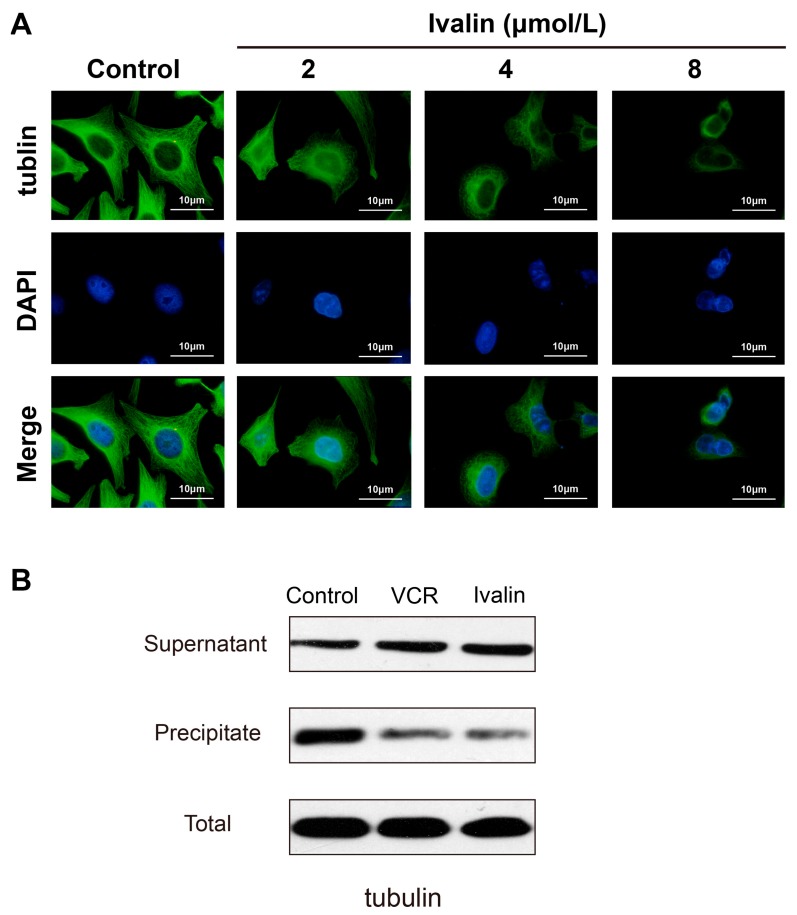
Ivalin destructed the normal microtubule structure in SMMC-7721 cells. (**A**) Cells were treated with Ivalin and analyzed by immunofluorescence staining; (**B**) Cells were treated with 4 μmol/L Ivalin or 0.1 μmol/L vincristine (VCR) for 24 h. After that, the soluble and the insoluble fractions of tubulin were separated and detected by Western blotting.

Immunofluorescence staining techniques were used to evaluate the effect of Ivalin on the cytoskeleton network in SMMC-7721 cells in order to investigate the mechanism underlying the G2/M arrest in Ivalin-treated cells. We treated the cells with various concentrations of Ivalin, ranging from 0 to 8 μmol/L, and the images acquired on fluorescent microscope are shown in Figure 5A. Compared to the typical cytoskeleton structures of untreated cells, which had long and dense microtubules extending throughout the cytoplasm, cells showed cellular microtubule depolymerization after 24 h of Ivalin treatment. These data indicated that Ivalin suppressed the cellular microtubule network formation by destructing the normal microtubule structure in SMMC-7721 cells.

If microtubules are depolymerizing, tubulin should shift from insoluble to soluble fraction. Consistently, treatment with Ivalin resulted in an induction of the depolymerizing tubulin (in supernatant) but a reduction of polymerized tubulin (in precipitate) in SMMC-7721 cells, without changing the level of total tubulin (Figure 5B).

## 4. Discussion

Highly dynamic mitotic-spindle microtubules are the long, filamentous, tube-shaped protein polymers that consist of α-tubulin and β-tubulin heterodimers [19]. Microtubules play an important role in maintaining cell morphology, transporting the components such as vesicles and mitochondria out of cells and carrying out the process of cell division and mitosis [5,20]. In this regard, a large number of chemically diverse microtubule-targeted compounds have been well used in clinical cancer treatment, making microtubules and their dynamics effective targets for anticancer therapies [21,22,23]. Most of the microtubule-targeted compounds, such as paclitaxel, colchicine, and vinblastine were discovered in natural plants [24,25,26,27,28]. Ivalin showed an excellent depolymerization effect on cellular microtubules in our present work. It is known that the depolymerization of microtubules disrupts the dynamic equilibrium and thus impairs the mitotic spindle assemble, which is considered crucial to successful mitosis. The cells will then remain blocked in a mitotic phase and eventually undergo proliferation inhibition or even programmed cell death such as apoptosis [29]. Our present results showed that Ivalin treatment induced a significant G2/M phase arrest in SMMC-7721 cells in a dose-dependent manner. The expression levels of Cdc25A and Cyclin B1 were increased after Ivalin treatment, while the expression of phosphor-Cdc2 and Cdc2 remained unchanged. It has been reported that the activation of M-phase promoting factors such as Cdc2/Cyclin B1 complexes and Cdc25 would initiate the transition from G2 to M phase [30,31]. Connecting all these phenomena, we suggested that Ivalin treatment led to SMMC-7721 cell arrest in the M-phase rather than the G2-phase after the depolymerization effect on cellular microtubule. However, the number of cells that arrested in G2/M-phase reached the maxima with 24 h of Ivalin treatment, decreasing during continuous treatment, indicating that subsequent apoptosis may occur because of the persistent mitotic arrest in SMMC-7721 cells.

B-cell lymphoma-2 (Bcl-2) family proteins, including pro-apoptotic (such as Bak and Bax) and anti-apoptotic (such as Bcl-xL and Bcl-2) proteins play important roles as critical checkpoints that regulate both intrinsic and extrinsic apoptosis [32]. Ivalin apparently reduced the expressions of Bcl-2 and induced that of Bax protein. In addition, flow cytometric analysis confirmed the apoptotic cells increased from 3.80% to 74.06% after being exposed to various concentration of Ivalin. As a result, we concluded that the normal microtubule structure in SMMC-7721 cells was destructed by inducing microtubule depolymerization after Ivalin treatment; then the cells were blocked in G2/M-phase, eventually resulting in apoptosis. However, it is noted that the cytotoxicity of Ivalin to normal human hepatocyte HL7702 cells was very low (25.86 ± 0.87 μmol/L), while a value of 4.34 ± 0.10 μmol/L was found for SMMC-7721 cells. It is known that mitosis progresses rapidly in cancer cells, which usually display higher rates of proliferation than normal cells. Microtubules are critical for cellular proliferation due to their important role in mitotic spindle apparatus constitution. At least one reasonable explanation for why normal cells are less sensitive to Ivalin than cancer cells is that there is a lower frequency of cell separation and therefore less of a chance of passing through a stage of exposure to mitotic poisons when compared with cancer cells. On the other hand, we speculate the possibility that Ivalin is a compound with multi-target activity. To confirm that, further research is required for Ivalin drug development.

## 5. Conclusions

In conclusion, the study presented a nature microtubule inhibitor Ivalin with potent anticancer activity. Ivalin induced microtubule depolymerization, then blocked cells in the mitotic phase and eventually resulted in apoptosis in SMMC-7721 cells. Ivalin could act as an effective leading compound for further development of anti-cancer drugs and as a prototype strategy for cancer therapy by targeting microtubules.

## Figures and Tables

**Table 1:** Growth inhibitory activities of Ivalin in different cell lines.

Cell Lines	IC_50_ (μmol/L)
SMMC-7721	4.34 ± 0.10
HepG-2	5.45 ± 0.13
Plc-prf-5	11.33 ± 1.00
Hu-7	13.01 ± 0.42
HL7702	25.86 ± 0.87

Ivalin exerted potent anti-proliferative activity on hepatocellular carcinoma cell lines. We evaluated the IC_50_ values (concentration resulting in 50% inhibition of cell growth) of Ivalin with different hepatocellular carcinoma (HCC) cell lines and normal human hepatocyte cell lines via 3-(4,5-dimethylthiazol)-2,5-diphenyltetrazolium bromide (MTT) assay after treatment with various concentrations of Ivalin.

## References

[1] Siegel R.L., Miller K.D., Jemal A. (2019). Cancer statistics, 2019. CA Cancer J. Clin..

[2] Abdalla E.K., Vauthey J. (2004). Focus on treatment of large hepatocellular carcinoma. Ann. Surg. Oncol..

[3] Xu Y., Qin X., Zhou J., Tu Z., Bi X., Li W., Fan X., Zhang Y. (2011). Tissue factor pathway inhibitor-2 inhibits the growth and invasion of hepatocellular carcinoma cells and is inactivated in human hepatocellular carcinoma. Oncol. Lett..

[4] Sun X., Li L., Ma H.G., Sun P., Wang Q.L., Zhang T.T., Shen Y.M., Zhu W.M., Li X. (2017). Bisindolylmaleimide alkaloid BMA-155Cl induces autophagy and apoptosis in human hepatocarcinoma HepG-2 cells through the NF-kappa B p65 pathway. Acta Pharm. Sin..

[5] Jordan M.A., Wilson L. (2004). Microtubules as a target for anticancer drugs. Nat. Rev. Cancer.

[6] Kavallaris M. (2010). Microtubules and resistance to tubulin-binding agents. Nat. Rev. Cancer.

[7] Mitchison T.J. (1988). Microtubule dynamics and kinetochore function in mitosis. Ann. Rev. Cell Biol..

[8] Rusan N.M., Fagerstrom C.J., Yvon A.M.C., Wadsworth P. (2001). Cell cycle-dependent changes in microtubule dynamics in living cells expressing green fluorescent protein-alpha tubulin. Mol. Biol. Cell.

[9] Jordan M.A., Thrower D., Wilson L. (1991). Mechanism of inhibition of cell proliferation by *vinca* alkaloids. Cancer Res..

[10] Schiff P.B., Fant J., Horwitz S.B. (1979). Promotion of microtubule assembly *in vitro* by taxol. Nature.

[11] Kaur R., Kaur G., Gill R.K., Soni R., Bariwal J. (2014). Recent developments in tubulin polymerization inhibitors: An overview. Eur. J. Med. Chem..

[12] Field J.J., Kanakkanthara A., Miller J.H. (2014). Microtubule-targeting agents are clinically successful due to both mitotic and interphase impairment of microtubule function. Bioorg. Med. Chem..

[13] Ganapathi R.N., Ganapathi M.K. (2013). Mechanisms regulating resistance to inhibitors of topoisomerase II. Front. Pharmacol..

[14] Perez E.A. (2009). Microtubule inhibitors: Differentiating tubulin-inhibiting agents based on mechanisms of action, clinical activity, and resistance. Mol. Cancer Ther..

[15] Ma J.H., Qi J., Liu F.Y., Lin S.Q., Zhang C.Y., Xie W.D., Zhang H.Y., Li X. (2018). Ivalin Inhibits Proliferation, Migration and Invasion by Suppressing Epithelial Mesenchymal Transition in Breast Cancer Cells. Nutr. Cancer Int. J..

[16] Xie W.-D., Wang X.-R., Ma L.-S., Li X., Row K.-H. (2012). Sesquiterpenoids from Carpesium divaricatum and their cytotoxic activity. Fitoterapia.

[17] Lin S., Zhang C., Liu F., Ma J., Jia F., Han Z., Xie W., Li X. (2019). Actinomycin V Inhibits Migration and Invasion via Suppressing Snail/Slug-Mediated Epithelial-Mesenchymal Transition Progression in Human Breast Cancer MDA-MB-231 Cells In Vitro. Mar. Drugs.

[18] Domingo-Sananes M.R., Kapuy O., Hunt T., Novak B. (2011). Switches and latches: A biochemical tug-of-war between the kinases and phosphatases that control mitosis. Philos. Trans. R. Soc. B Biol. Sci..

[19] Luduena R.F., Jeon K.W. (1997). Multiple forms of tubulin: Different gene products and covalent modifications. International Review of Cytology—A Survey of Cell Biology.

[20] Nogales E., Stroud R.M., Olson W.K., Sheetz M.P. (2001). Structural insights into microtubule function. Annual Review of Biophysics and Biomolecular Structure.

[21] Jordan M.A., Wilson L. (1998). Microtubules and actin filaments: Dynamic targets for cancer chemotherapy. Curr. Opin. Cell Biol..

[22] Giannakakou P., Sackett D., Fojo T. (2000). Tubulin/microtubules: Still a promising target for new chemotherapeutic agents. J. Natl. Cancer Inst..

[23] Wilson L., Jordan M.A. (1995). Microtubule dynamics: Taking aim at a moving target. Chem. Biol..

[24] Podolski-Renić A., Banković J., Dinić J., Ríos-Luci C., Fernandes M.X., Ortega N., Kovačević-Grujičić N., Martín V.S., Padrón J.M. (2017). DTA0100, dual topoisomerase II and microtubule inhibitor, evades paclitaxel resistance in P-glycoprotein overexpressing cancer cells. Eur. J. Pharm. Sci..

[25] Yi J.M., Zhang X.F., Huan X.J., Song S.S., Wang W., Tian Q.T., Sun Y.M., Chen Y., Ding J., Wang Y.Q. (2015). Dual targeting of microtubule and topoisomerase II by alpha-carboline derivative YCH337 for tumor proliferation and growth inhibition. Oncotarget.

[26] Horwitz S.B. (1994). How to make taxol from scratch. Nature.

[27] Hastie S.B. (1991). Interactions of colchicine with tubulin. Pharmacol. Ther..

[28] Na G.C., Timasheff S.N. (1980). Thermodynamic linkage between tubulin self-association and the binding of vinblastine. Biochemistry.

[29] Jordan M.A., Wendell K., Gardiner S., Derry W.B., Copp H., Wilson L. (1996). Mitotic block induced in HeLa cells by low concentrations of paclitaxel (Taxol) results in abnormal mitotic exit and apoptotic cell death. Cancer Res..

[30] Duesbery N.S., Vande Woude G.F. (1998). Cytoplasmic control of nuclear behavior during meiotic maturation of frog oocytes. Biol. Cell..

[31] Doree M., Labbe J.C., Picard A. (1989). M phase-promoting factor: Its identification as the M phase-specific H1 histone kinase and its activation by dephosphorylation. J. Cell Sci..

[32] Cheng E. (2009). Molecular Control of Mitochondrial Apoptosis by the BCL-2 Family. Blood.

